# A Patient Navigator Intervention Supporting Timely Transfer Care of Adolescent and Young Adults of Hispanic Descents Attending an Urban Primary Care Pediatrics Clinic

**DOI:** 10.1097/pq9.0000000000000391

**Published:** 2021-03-10

**Authors:** Sophie Allende-Richter, Patricia Glidden, Mariam Maloyan, Zana Khoury, Melanie Ramirez, Kitty O’Hare

**Affiliations:** From the * Division of General Pediatrics, Department of Medicine, Boston Children’s Hospital, Boston, Mass.; † Department of Pediatrics, Harvard Medical School, Boston, Mass.; ‡ Tufts University, Department of Community Health, Boston, Mass.; § Department of Medicine, Harvard Medical School, Boston, Mass.; ¶ WakeMed Children’s Hospital, Raleigh, N.C.

## Abstract

While comprehensive health care transition is associated with better health outcomes, navigating health care transition can be difficult for adolescents and young adults (AYAs), especially those with fewer resources. Our practice serves low-income patients from birth to their 26th birthday; many are medically and socially complex and experience several obstacles to navigate care. As a result, most have not initiated a transfer to adult medicine by age 25. This quality-improvement initiative was designed to implement a structured intervention that supports the planned transfer of care to adult primary care.

**Methods:**

Informed by our baseline data on all patients eligible to transfer care, we designed a patient outreach workflow centered on a patient navigator (PN) intervention. We used a Plan-Do-Study-Act format to optimize our process and run charts to evaluate our intervention.

**Results:**

Over 3 years, our PN reached out to 96% of patients (n = 226) eligible to transfer care and offered transfer assistance in person or in writing. Among those surveyed, 92% (n = 93) reported awareness of our practice transition policy, and 83% (n = 64) rated their confidence to transfer care at 3 or higher on a 5-point scale.

**Conclusions:**

AYAs are aware of our practice transition policy, yet they welcome in-person transfer assistance. This intervention seems to improve their confidence to transfer care. However, despite PN outreach efforts, many remain empaneled in our practice and thus lack the self-care skills necessary to complete the transfer independently. Future transition interventions should address AYA’s self-management skills toward transition readiness.

## Abstract 2

Supplemental Digital Content is available in the text.

## INTRODUCTION

Our primary care practice, located in a low-income neighborhood of Boston, serves patients from birth to their 26th birthday; most are of Hispanic descent and qualify for public insurance (Medicaid). The complexity of their medical and psychosocial needs creates barriers and challenges when navigating healthcare systems. As a result, many are unprepared to transition to adult care at age 25. Failure to transition to adult care is associated with an increased demand on pediatric providers untrained in adult medicine,^1^ gaps in healthcare access,^1–4^ preventable emergency department visits, and hospital admissions among young adults.^3,4^ The process of transferring care of adolescent and young adult (AYA) patients from pediatric to adult primary care is formally known as healthcare transition (HCT). It is critical to ensure that AYAs maintain access to high-quality and developmentally appropriate care at a time when they are particularly vulnerable due to psychological, physical, and social changes, including changes in health insurance coverage.^5,6^ Poorly executed HCT leads to poor treatment adherence,^7^ lapse in health insurance coverage,^8^ gaps in healthcare access,^1,2^ and overall poor health outcomes.^9^ While research on transition efforts have mostly centered around youth with special health care needs (YSHCN),^10–12^ a recent study reports low levels of comprehensive transition provision among youth with (17%) and without (14%) special health care needs across the United States.^13^ AYAs of underserved backgrounds are especially at risk for poor transition preparation due to multiple logistical and financial obstacles to access care, housing insecurity, and low education attainment among their parents.^14–16^

### Transition Framework

In 2011, the American Academy of Pediatrics, American Academy of Family Physicians, and American College of Physicians-American Society of Internal Medicine issued a clinical report providing a framework to support the HCT process from adolescence to adulthood in 6 steps. These steps are the 6 core elements of HCT. They include the following:

(1) Transition policy
(2) Transition tracking and monitoring
(3) Transition readiness
(4) Implementation of processes for transition planning
(5) Transfer of care
(6) Transfer completion and documentation.^17,18^

These six core elements were intended to guide healthcare professionals to ensure that AYA patients receive adequate support toward their transfer to adult care.^18^ Despite these guidelines, there is a lack of an explicit model of transition intervention.^13–19^ Nationwide low rates of transition planning and widening health disparities among AYAs warrant further interventions to ensure timely and successful transfer to adult medicine.^13–19^

### Patient Navigator

Several publications indicate that patient navigator (PNs) intervention around care coordination improves healthcare access among medically complex and/or socioeconomically disadvantaged patient populations.^20,21^ One intervention involving PNs among type I diabetic patients resulted in increased medical follow-up rates, decreased hospital admissions, and overall better health outcomes.^22,23^ With over 20% of AYA patients reaching the upper age limit to transfer care per our practice transition policy and increasing demands on providers to complete multiple and complex tasks within the limited time provided during a visit, we decided to implement a PN transfer outreach intervention to track and provide transfer assistance to empaneled patients who met criteria to transfer care.

#### Specific Aims

We adopted the following aims.

1. At least 50% of patients eligible to transfer care and scheduled for an annual health examination will receive in-person transfer assistance at the time of their visit.
2. The PN will reach out to 90% of patients eligible to transfer care in person or in writing to notify them of our transition policy and offer transfer assistance.
3. A minimum of 50% of patients who received in-person transfer assistance will rate their confidence to transfer care at 3 or higher on a 5-point scale.

## METHODS

### Setting

The setting is a single-site urban academic pediatric primary care practice located in a low-income neighborhood of Boston, serving children, adolescents, and young adults through their 26th birthday.

Seventy-six percent of the practice population self-identifies as Hispanic; the majority are first- or second-generation immigrants. Ninety percent qualify for public insurance (Medicaid). Twenty-three percent of the total patient population is of adult age (18+), with 20% of those being of transfer age (24–26) per our practice transition policy. The practice is composed of 2 care teams: one focused on pediatric patients of ages 0–13 years and the other on adolescent patients age 13 years and above. Each group includes medical providers, nurses, social workers, clinical and administrative assistants, a nutritionist, and a Spanish-speaking PN. Patients typically transfer care from the pediatric care team to the adolescent team between 13 and 15 years, where they are cared for by adolescent medicine-trained clinicians.

### Intervention

#### Planning of the Intervention

A review of the adolescent care team’s patient panel in our electronic health record (EHR) demonstrated that the care team followed many patients within the transfer age range. After reviewing the empanelment data, our practice leadership allocated up to 3 hours a week or 7.5% full-time equivalent of an existing administrative staff position toward the PN role and about 2 hours a week of a practice data coordinator time to support a HCT quality improvement (QI) intervention. The cost of office supply generated by this study was absorbed within our practice operation budget. No additional staff was hired for this study.

**Fig. 1.**
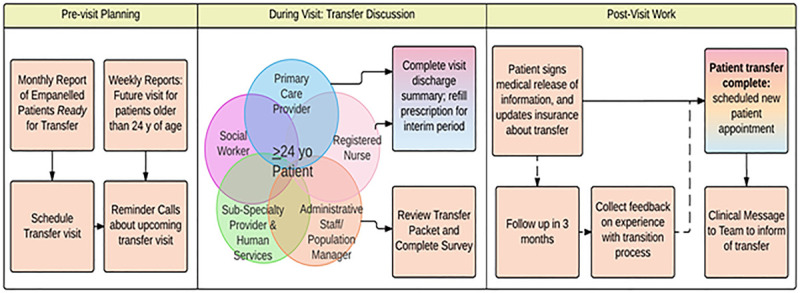
Patient navigator transfer outreach and care coordination workflow. The above workflow provides a visual description of the different components needed to support this process: previsit planning, multidisciplinary involvement, and postvisit follow-up to confirm the transfer.

We designed a PN Transfer outreach workflow (Fig. 1) centered on a PN intervention to identify AYA patients eligible to transfer care and provide transfer assistance. A weekly report of patients who met the criteria to transfer care was autogenerated to support this process. For each patient listed, it informed the PN of any incoming appointment, patient primary care provider (PCP) name, and date of last physical examination. Also, we created a transfer package of information that included a brief transition policy statement, a directory of local adult primary care practices, a release of medical health information form, and a summary of 3 action steps needed for patients to complete transfer of care: (1) identification of a new adult provider; (2) update health insurance with new provider information, and (3) transfer of medical record. The PN role was to (1) assess patients’ transition policy awareness, transfer status, awareness of steps to complete transfer of care; (2) provide transfer assistance when necessary using the transfer package to educate patients about the transfer process, and (3) assess their confidence to transfer care as a result of this intervention (see table 1, Supplemental Digital Content, which displays Patient Navigator Survey Questions, http://links.lww.com/PQ9/A237).

#### Improvement Team

This QI intervention was led by a multidisciplinary team, which included 2 medical providers, a PN, a data coordinator, and a HCT physician consultant.

#### Study of the Intervention

The implementation phase began in July 2015. A study of the intervention took place from September 2015 to December 2018. We used a Plan-Do-Study-Act (PDSA) cycle approach to optimize PNs outreach efforts and disseminate our transition policy across the entire practice to improve patient and staff awareness around HCT. Our improvement team met at least monthly to evaluate the intervention. We surveyed patients to assess their awareness of our transition policy and self-confidence to transfer care. We surveyed our staff to evaluate their confidence to provide transfer assistance to patients.

#### Measures

##### Process Measures

1. The percentage of patients scheduled for an annual health examination and eligible to transfer care that the PN met in person and provided a package of transfer information.
2. When unable to meet with patients in person, as a back-up measure, the PN mailed a postcard (see figure, Supplemental Digital Content 1, which displays percent of 24- to 25-year-old patients notified of our transition policy by mail or in person at their annual health examination, http://links.lww.com/PQ9/A236 and table 2, Supplemental Digital Content, which displays transfer postcard notification, http://links.lww.com/PQ9/A238) to each patient due for transfer of care, notifying them of our transition policy and offered to schedule transition assistance in clinic.

##### Outcome Measures

1. The percentage of patients scheduled for an annual health examination and eligible to transfer care who received PN transfer assistance and reported awareness of our transition policy;
2. The percentage of patients who received PN transfer assistance reporting confidence in their ability to transfer care greater than 3 on a 5-point confidence scale.

#### Analysis

We used quantitative and qualitative approaches through iterative PDSA cycles to evaluate the intervention. Our improvement team met regularly during this process and provided feedback related to our transition process intervention. We conducted several PDSA cycles to determine the best means of outreach to this group. Data were analyzed using descriptive statistics. We used SQCpack 7 (PQ Systems, Dayton, OH), a data analysis software, to create run charts, track changes over time, and evaluate outcomes. The following run chart rules identify a change based on *The Data Guide*: (1) a shift is defined as 6 or more consecutive points that all fall above or below the mean line; (2) a trend as 5 or more successive points all going up or down; (3) a run as a series of consecutive points that form a nonrandom pattern by which too few runs cross the mean.^24^ We annotated each PSDA on the charts to identify the intervention’s impact on the data series. Our data collection took place from Q3 2015 at the start of the first intervention and continued through the study period. Select PDSA cycles required their data collection schedule, which maintained isolated periods of data collection and analysis.

#### Ethics

Our department approved this intervention as a QI initiative and thus was exempted from review by the hospital institutional review board. All data collected were anonymously stored and strictly used for our process evaluation.

## RESULTS

**Fig. 2.**
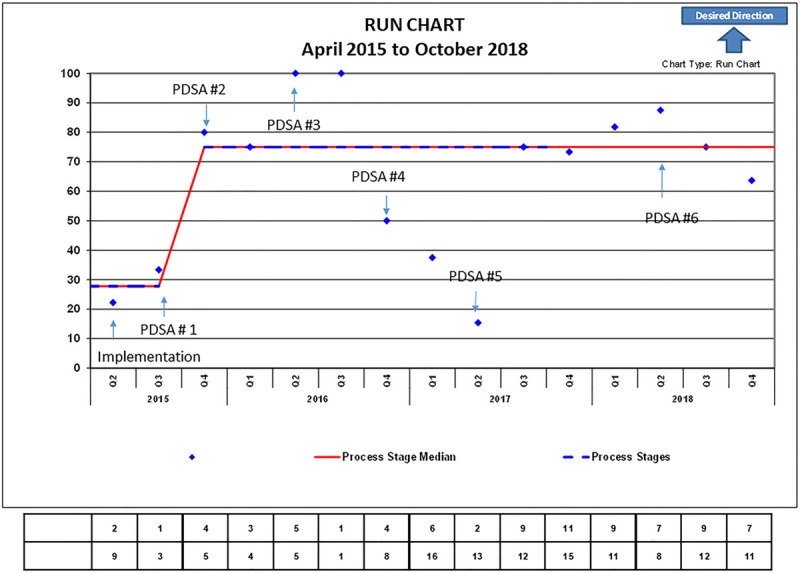
Patient navigator transfer assistance outreach efforts run chart. The above graph describes the percentage of patients who met with patient navigator at 24- and 25-year-old physical (y axis) from Q2 2015–Q4 2018 on the (x axis). *Baseline data point based on outreach and scheduled visits with the patient navigator. **Inclusion of 24-year-old physicals in the total number of visits.

The following results describe the test of changes we implemented to support our transfer assistance intervention. At baseline, we identified 94 actively empaneled patients of age 25 years and older from our EHR. Our PN conducted initial outreach efforts by phone and contacted 25 (27%) patients successfully. Of those 25 patients, 15 (60%) completed the phone interview and 6 of 15 (40%) of them reported to have transferred care to an adult primary care physician while 9 of 15 (60%) had not transferred. Of the 9 patients who had not transferred care, 8 patients reported awareness of our practice transition policy, but most (data not recorded) reported low confidence to transfer care 3 or less on a 5-point scale. All 9 patients requested to be scheduled for a transfer visit to complete. Of those 9 patients, only 2 (22%) met the PN in the clinic and received transfer assistance (Fig. 2, baseline).

We quickly learned that it was logistically challenging and time-consuming to provide transfer assistance by phone from this implementation phase. Therefore, the PN would meet patients in the clinic during their scheduled appointment to assess transfer status and provide transfer assistance. This change led to a 10% increase from the baseline in the number of patients who received PN assistance (Fig. 2, Q3 2015; Table 1). To optimize in-clinic transfer assistance, the PN reviewed a weekly autogenerated electronic report of actively empaneled patients eligible to transfer with an incoming appointment and called each patient before their appointment (Fig. 2, PDSA 1; Table 1). As a result of this intervention, our PN could meet 80% of patients scheduled at their annual 25-year-old physical during this quarter and provide them with transfer assistance (Fig. 2, Q4 2015). At times it was still challenging for the PN to meet consistently with each patient in person due to logistical challenges with clinic workflow and PN competing tasks, leading to a slight drop in the percentage of patient outreach to 75% (Fig. 2, Q1 2016), which lead us to plan our second test of change to sustain in-clinic PN outreach efforts. We informed our front-desk staff of our process and tasked them to notify the PN when patients checked-in and provide basic transfer assistance should the PN have scheduling conflicts to meet with patients (Fig. 2, PDSA 2; Table 1). Although the PN was subsequently able to meet 100% of patients scheduled in the clinic, surpassing our goal line of 50%, the number of patients receiving transfer assistance remained relatively low in contrast with the overall volume of patients 25-year-old and older still actively empaneled (Fig. 2, Q2–Q3 2016).

**Table 1.** PDSA Cycles 1-6 Patient Navigator Transfer Outreach Intervention

Q2 2015	Implementation	Plan: Reach out to empaneled patients older than 25 years of age to assess transfer status
		Do: Launch transfer registry; PN calls patients
		Study: Patients aware of need to transfer, but have not initiated process and welcome transfer
		Act: PN to meet patients in-person during appointment
Q3 2015	PDSA 1	Plan: PN meets patients in clinic and provide in-person transfer assistance
		Do: PN reviews weekly report of scheduled patients, calls patients to remind appointment, meet patients in clinic to assess awareness of transition policy, transfer status, need for transfer assistance, provides transfer packet of information and assess confidence to transfer care
		Study: Patients welcome assistance, modest outreach due to logistical challenge
		Act: Informed front desk staff of transfer process
Q1 2016	PDSA 2	Plan: Optimize in-clinic outreach
		Do: Front desk staff informed of transfer process, notifies PN when patient checks-in, provides transfer package if PN unavailable, communicate with PN, PN closes loop on transfer completion
		Study: Increase percentage of patient receiving transfer assistance, intervention limited to in-clinic only, does not account for missed or unscheduled patients.
		Act: Broaden PN outreach efforts
Q2 2016	PDSA 3	Plan: All patients to be notified of need to transfer care at age 25 in writing or verbally
		Do: Mailing of transfer post-card to patient turning 25, dissemination of Transition Policy, statement provided to patients at each physical starting at age 18. Tracking of returned post-card mailed
		Study: about 98% of patient 25 years older were informed of transfer assistance; this also resulted in an increase in volume of 25 years old patient receiving transition assistance in clinic
		Act: Inform all staff across clinic about Transfer process to direct demand of patient seeking transfer assistance.
Q4 2016	PDSA 4	Plan: Assess staff awareness of transition policy and interventions
		Do: Conduct staff survey
		Study: Low level of staff awareness but willingness to learn more about Transition
		Act: 20-minute presentation at all staff meeting on Health Care Transition and ongoing transition intervention
Q2 2017	PDSA 5	Plan: Expand target age to include 24-year-olds to allow adequate time to transfer care
		Do: Modified existing electronic report to include patients from age 24 years, PCP, problems list PN to use this report and generated a transfer checklist message to notify by clinical message the care team of transfer assistance needs for patient age 24 and above. Transfer checklist also posted in clinical area and at huddle to delegate care team to actively engaged in transfer planning by skill set.
		Study: Multidisciplinary approach increases percentage of patients receiving in clinic transfer assistance and leads to an upward shift of the mean.
		Act: Additional admin staff added to PN team to provide back-up transfer assistance as needed.
Q1 2018	PDSA 6	Plan: Sustained volume of patient receiving in-person assistance.
		Do: Training of 2 admin staff PN back-up to meet increase volume in in-person transfer assistance.
		Study: Initial increase in-person assistance followed by a slight decrease due to staffing change without affecting mean and goal.

To account for patients who did not receive transfer assistance in the clinic, the PN mailed a transfer notification postcard (see table 2, Supplemental Digital Content, which displays Transfer postcard notification, http://links.lww.com/PQ9/A238) monthly to each patient who turned 25 that month and had not received in clinic transfer assistance, to notify them of our practice transition policy and offer in-person PN transfer assistance (Fig. 2, PDSA 3; see figure, Supplemental Digital Content 1, which displays percent of 24- to 25-year-old patients notified of our transition policy by mail or in person at their annual health examination, http://links.lww.com/PQ9/A236). This process was added to the registry as well as any returned mailed postcard. The clinic leadership approved the posting of the practice transition policy statement in all clinical areas and its distribution to patients at each annual health examination visit starting from 18 years.

As a result of this intervention, 98% of 25-year-old patients received a transfer notification in person or by mail (figure, Supplemental Digital Content 1, which displays percent of 24- to 25-year-old patients notified of our transition policy by mail or in person at their annual health examination, http://links.lww.com/PQ9/A236). The transfer notification postcard did not immediately generate an increase in the 25-year-old patients scheduled for an annual health examination (Fig. 2, Q3 2016). To ensure that staff appropriately directed patients at any checkpoint in and around the clinic (ie, patient call center), we conducted a staff survey (Table 2) to assess staff awareness of our transition practice policy and interventions. The survey results revealed a low level of awareness of our practice transition policy and existing transition intervention among our staff, yet 56% of the survey respondents (n = 25) expressed interest in training on this process (Table 2). To this effect, our QI team leader gave a 20-minute presentation on HCT and ongoing practice intervention during a staff meeting (Fig. 2, PDSA 4). Although those later efforts contributed to the increase in the volume of 25-year-old patients scheduled for an annual health examination, we noted that the PN was not able to meet the increased volume of patients seeking in-person transfer assistance (Fig. 2, Q4 2016–Q2 2017; Table 1). We identified our efforts to be reactive, leaving very little time and opportunity for 25-year-old patients to prepare adequately to transfer care if they did not receive assistance in the clinic. In response, we adopted a more proactive approach by expanding our target outreach age to include patients from the age of 24 and implementing a multidisciplinary team approach. We modified the preexisting report to include 24-year-old patients; we also added a column listing any chronic medical diagnoses and previous social work or mental health encounters.

**Table 2.** Transition Policy Staff Survey (October 2016)

Transition Policy Staff Survey Responses (n = 25)	n (%)
I am a/an	
Attending	5 (20%)
Resident	2 (8%)
RN	4 (16%)
Other team member	14 (56%)
Are you familiar with the Martha Eliot Transition Policy?	
Yes	14 (56%)
No	11 (44%)
How important do you think it is to have and post a Transition Policy in your clinical area?	
1 (not important)	0 (0%)
2 (neutral)	0 (0%)
3 (somewhat important)	6 (24%)
4 (very important)	9 (36%)
5 (extremely important)	10 (40%)
Do you feel comfortable assisting a patient around transition in your clinical area?	
Yes	10 (40%)
Somewhat comfortable	6 (24%)
No	9 (36%)
Would you be interested in more training around transition of care?	
Yes	14 (56%)
Neutral	7 (28%)
No	4 (16%)

To optimize in-clinic transfer assistance, the QI team reviewed this report monthly to identify proactive areas where patients would need the most assistance and generate a transfer checklist to be completed by the care team. The PN subsequently sent this transfer checklist as a clinical message in the patient EHR to the PCP and entire care team ahead of patient appointment (see table 3, Supplemental Digital Content, which displays transfer checklist clinical message, http://links.lww.com/PQ9/A239). We posted a template of the transfer checklist in each provider office and nursing station to elicit transfer planning discussion during huddle and involve each care team (nursing, social worker, administrative assistance, PCP, PN) to complete these tasks according to their skillset toward the transfer of care (Figs. 1, 2, PDSA 5; Table 1). Our intervention’s proactive approach immediately improved the percentage of patients of age 24+ who received in-clinic transfer assistance leading to a shift of our mean from 44% to 78% (Fig. 2, Q2–Q3 2017). To meet the volume increase of patients in need of transfer assistance, we trained 2 additional administrative staff to provide back-up transfer assistance on an as-needed basis without increasing PN hours of involvement (Fig. 2, PDSA 6; Table 1). However, after an initial increase in the percentage of patients receiving in-clinic transfer assistance, we noticed a slight volume decrease reflecting a staffing change in our PN pool. Nevertheless, these changes did not negatively impact our mean, and we were able to remain above our goal line of 50% for the subsequent quarters (Fig. 2, Q3–Q4 2018).

Over 3 years, our PN contacted 218 (96%) patients of 226 eligible to transfer care, informed them of our practice transition policy, and offered transfer assistance. Of those 218 patients, 102 (48%) received in-clinic transfer assistance, and 116 (52%) were offered transfer assistance in writing by the mean of a transfer postcard notification. Ninety-two percent approached by the PN (n = 86/93) reported awareness of our practice transition policy, and 83% (n = 53/64) of patients rated confidence to transfer at 3 or higher on the 5-point Likert scale. Patients who rated themselves at 3 or higher were considered confident in their abilities to complete the transfer of care consistent with our aims.

## DISCUSSION

The authors acknowledge that due to the lack of robust baseline data, outcomes for this study are strictly observational due to the intervention’s novelty. Nevertheless, we learned from our implementation phase that most patients who had not yet transferred care were aware of our practice transition policy (8 of 9) had a low level of confidence in their ability to transfer care of 3 or less on a 5-point scale. Since all 9 patients welcomed in-person transfer assistance, we assumed that most, if not all, had a low level of confidence to transfer care independently. In-clinic transfer outreach proved to be more successful than phone outreach (Fig 2. Q4, 2015–Q4, 2018). We explained deflections in our in-clinic transfer assistance due to logistical challenges such as missed appointments, PN competing tasks, or clinic workflow impeding in-person transfer assistance (Fig. 2 Q1 2016; Q4 2016–Q2, 2017). We were able to address these by increasing our administrative staff awareness of our transition policy, through training and mailing of transfer notification postcards (Fig. 2 PDSA No. 2–4). However, despite those small tests of change, we were only able to reach a larger fraction of patients in the clinic and meet consistent results once we adopted 2 strategies, which include providing transfer assistance 1 year earlier than their 25th birthday to allow for enough planning time and a proactive multidisciplinary team approach (Figs. 1, 2 Q3 2017–Q4 2018).

Although the primary purpose of the multidisciplinary team involvement in this transfer process was to provide clinical support around the transfer of care, we believe that the fact that patients were identified proactively and this was communicated to the care team created a safety net, increasing the likelihood that a given patient would meet with the PN and receive transfer assistance while in the clinic. Ultimately, it is a proactive approach, supportive leadership, monthly multidisciplinary team meetings, administrative staff involvement, and multimodal efforts toward patient engagement, allowing us to reach our goals and establish a sustainable transfer process (Figs. 1, 2).

Due to a growing number of AYAs reaching the upper age limit to transfer care, we made the deliberate choice to focus our intervention on the transfer of care, the fifth step of the 6 core elements of HCT. However, we acknowledge the lack of transfer outcome data as a significant weakness in our process validation. Early in our implementation phase, we reached out to local adult practices to identify which practice accepted new patients and informed them of our process. However, transfer tracking and documentation of transfer completion (the sixth step of the 6 core elements) were probably our most significant limitations as the patients owned part of this step. We noted that one of the major limiting factors for patients was registering in a new adult practice and notifying their health insurance of the PCP change independently. This issue seems to be a daunting task for many, as it required calling their health insurance, and in many instances, this meant long waiting time until one could receive assistance. The PN could only assist the most medically complex patients with this step and had to rely on what became a lengthy and time-consuming list of patients follow-up calls to confirm transfer completion. Despite a relatively successful outreach effort, we only observed a modest decline, 10% average (data not shown), in the number of actively empaneled patients eligible to transfer care, over 3 years. We believe that allowing patients to remain in a pediatric primary care practice until their young adult age of 25 years may hinder their ability to transfer care timely. These observations led us to believe that despite reporting confidence to transfer care, our AYAs lack the self-management skills necessary to complete their transfer to adult medicine.

## CONCLUSIONS SUMMARY

Our primary goal in sharing these results is to provide the medical community with the practical steps to implement a structured transfer process, which we felt is much needed. While the intervention described may seem relatively complicated, we believe that the key to its success relied on leadership support and administrative staff training and should be reproducible at a relatively low cost. Future transition interventions should involve accountable care organizations to strengthen collaboration with adult practices and ensure transfer completion. The authors also believe that the early dissemination of a transition policy across practice is key to successful care transfer. We also recommend the early introduction of software technology, such as patient portal application, to promote self-management skills as soon as developmentally appropriate.^19,25^

## DISCLOSURE

The authors have no financial interest to declare in relation to the content of this article.

## ACKNOWLEDGMENTS

We thank the Boston Children Hospital Primary Care Administrative and Clinical Leadership for their outstanding support.

## Supplementary Material


