# Supplementary material for: A Patient Navigator Intervention Supporting Timely Transfer Care of Adolescent and Young Adults of Hispanic Descents Attending an Urban Primary Care Pediatrics Clinic
Source: Pediatr Qual Saf. 2021 Mar 10;6(2):e391. doi: 10.1097/pq9.0000000000000391 (PMC7952101; doi:10.1097/pq9.0000000000000391)

Figure A. Percent of 24-25 years old patients notified of ourTransition policy by mail or in-person at their annual health exam

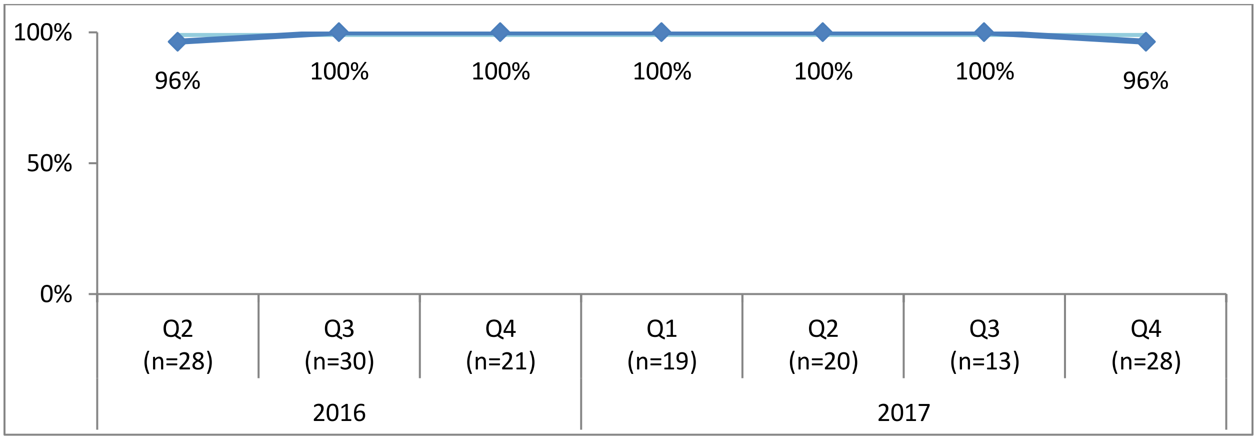

Supplement: Supplementary file 1 [file pqs-6-e391-s001.pdf]
