# Supplementary material for: A Patient Navigator Intervention Supporting Timely Transfer Care of Adolescent and Young Adults of Hispanic Descents Attending an Urban Primary Care Pediatrics Clinic
Source: Pediatr Qual Saf. 2021 Mar 10;6(2):e391. doi: 10.1097/pq9.0000000000000391 (PMC7952101; doi:10.1097/pq9.0000000000000391)
Supplement: Supplementary file 2 [file pqs-6-e391-s002.pdf]

SDC Table A. Patient Navigator Survey Questions

1. Aware of Transfer policy?<br>2. Have you Transferred your Care?	
Yes	No
1. New Clinic Name<br>2. Did you change Health Insurance?<br>3. Have you provided us with a signed written release of medical info?<br>4. How prepared do OR did you feel to complete your transfer? Scale 1 - 5	1. Would you like to make another visit with PCP?<br>2. Did you receive a Transfer information Packet?<br>3. Have you provided us with a signed written release of medical info?<br>4. How prepared do feel to complete the steps in your transfer process? Scale 1 – 5
Was this intervention helpful to you?	
