# Supplementary material for: A Patient Navigator Intervention Supporting Timely Transfer Care of Adolescent and Young Adults of Hispanic Descents Attending an Urban Primary Care Pediatrics Clinic
Source: Pediatr Qual Saf. 2021 Mar 10;6(2):e391. doi: 10.1097/pq9.0000000000000391 (PMC7952101; doi:10.1097/pq9.0000000000000391)
Supplement: Supplementary file 3 [file pqs-6-e391-s003.pdf]

SDC Table B. Transfer postcard notification

Dear [Patient Name],<br><br>Our records indicate that you recently turned 25 years old. Our Clinic would like to thank you for choosing us as your primary care provider. <u>We provide care our patients up to their 26th birthday.</u> Therefore, it is time for you to begin the process of transferring care to an adult primary care provider. We want to assist you in the transfer process!<br><br>If you have already transferred, please call us so we can update our records.<br><br>Please call (617) 971-2100 TODAY to schedule an appointment with your CLINICIAN TO DISCUSS THE TRANSFER OF YOUR CARE.<br><br>If we do not hear back from you <u>within 3 months</u> after you have received this letter, we will assume that you have transferred care and will update our record accordingly<br><br>Thank you for allowing us to serve you,
