# Supplementary material for: A Patient Navigator Intervention Supporting Timely Transfer Care of Adolescent and Young Adults of Hispanic Descents Attending an Urban Primary Care Pediatrics Clinic
Source: Pediatr Qual Saf. 2021 Mar 10;6(2):e391. doi: 10.1097/pq9.0000000000000391 (PMC7952101; doi:10.1097/pq9.0000000000000391)
Supplement: Supplementary file 4 [file pqs-6-e391-s004.pdf]

SDC Table C. Transfer Checklist Clinical Message[1]

**<u>For ALL patients, are there any:</u>**<br>[ ] Ongoing specialty care: if yes, seek assistance for adult specialty referral if indicated<br>[ ] Ongoing mental health services: if yes, notify social worker to assist w/ transition planning<br>[ ] Chronic medication: if yes, communicate w/ RN for patient education around med refill<br>[ ] Insurance issues: If yes, PER to provide assistance and direct patient to proper service<br>**<u>For KASA* patient:</u>**<br>**KASA care team will verify and update patient and yourself on the following:**<br>[ ] Patient has SSI benefits, FSN/DDS<br>[ ] Formal plan in place for adult living/vocation<br>[ ] Guardianship<br>**<u>For All patients:</u>**<br>[ ] Meet with Population manager at the end of the visit to provide assistance with transfer of care<br>[ ] schedule a 6 month f/up to address pending medical need and f/up on transfer readiness<br><br>**<u>For 25 years old patients,</u>**<br>New adult clinic: ___________________________<br>New adult PCP: _____________________________<br>Active Insurance: ______________________<br>Adult specialty care or mental health services: __________________

[1] **KASA** is an acronym for **K**ids and **A**dolescent with **S**pecial **A**bility and is a terminology our practice has adopted to flag special attention to patient with special health care needs.
